# Prevalence of ESBL-Producing Enterobacteriaceae in Pediatric Bloodstream Infections: A Systematic Review and Meta-Analysis

**DOI:** 10.1371/journal.pone.0171216

**Published:** 2017-01-31

**Authors:** Myrto Eleni Flokas, Styliani Karanika, Michail Alevizakos, Eleftherios Mylonakis

**Affiliations:** Infectious Diseases Division, Warren Alpert Medical School of Brown University, Rhode Island Hospital, Providence, RI, United States of America; Centre Hospitalier Universitaire Vaudois, FRANCE

## Abstract

**Background:**

Pediatric bloodstream infections (BSIs) with Extended-Spectrum Beta-Lactamase- producing Enterobacteriaceae (ESBL-PE) are associated with worse clinical outcomes. We aimed to estimate the prevalence of and the mortality associated with ESBL-PE in this patient population.

**Methods:**

A systematic review and meta-analysis using PubMed and EMBASE and included studies reporting the prevalence of ESBL-PE among confirmed BSIs in patients <19 years old.

**Results:**

Twenty three (out of 1,718 non-duplicate reports) studies that provided data on 3,381 pediatric BSIs from 1996 to 2013 were included. The prevalence of ESBL-PE was 9% [95%CI (6, 13)] with an annual increase of 3.2% (*P* = 0.04). The prevalence was 11% [95%CI (6, 17)] among neonates, compared to 5% [95%CI (0, 14)] among children older than 28 days. The pooled prevalence was 15% in Africa [95%CI (8, 23)], 12% in South America [95%CI (5, 23)], 11% in India [95%CI (7, 17)], 7% in the rest of Asia [95%CI (0, 22)], 4% in Europe [95%CI (1, 7)] and 0% in Oceania [95%CI (0, 3)]. Importantly, the mortality in neonates with BSI due to ESBL-PE was 36% [95%CI (22, 51)], compared to 18% [95%CI (15, 22)] among all other neonates with BSI and this difference was statistically significant (*P* = 0.01).

**Conclusions:**

In the pediatric population, the prevalence of BSI due to ESBL-PE is significant and is associated with increased mortality in neonates. Further studies are warranted to establish a high-risk group and the evaluation of preventive measures, such as antibiotic stewardship programs and infection control measures, in this population is urgently needed.

## Introduction

Extended-spectrum beta-lactamases (ESBL) are enzymes produced by Enterobacteriaceae that hydrolyze most beta-lactams [1] They are frequently encoded by plasmids that carry genes conveying resistance to other antibiotic groups, such as aminoglycosides and fluoroquinolones [1]. According to the 2013 report of the Centers for Disease Control and Prevention, ESBL-producing Enterobacteriaceae (ESBL-PE) were classified as a serious threat [2] and the prevalence of ESBL infections keeps rising [1,3]. In the pediatric population, bloodstream infections (BSIs) with ESBL-producing Enterobacteriaceae (ESBL-PE) are associated with longer hospital stays, increased healthcare costs and worse outcomes [4–7]. For example, the SENTRY Antimicrobial Surveillance Program in Europe, North and South America (1997–2002) reported that among <1 and 1–12 years old children, the prevalence of ESBL-producing isolates among *Klebsiella* spp. bloodstream pathogens was 41.7% and 31.3%, respectively [8].

Carbapenems are the mainstay of treatment of BSIs caused by ESLB-PE [9], and are not part of the established empiric therapy in most areas. Moreover, among adults with BSI due to ESBL-PE, failure to provide adequate antibacterial therapy within 72 hours of infection is an independent risk factor for mortality [10]. Given the clinical significance of these infections, we aimed to evaluate the burden of ESBL-PE among pediatric BSIs. More specifically, the purpose of this systematic review and meta-analysis is to estimate the prevalence and geographical distribution of BSI attributed to ESBL-PE among pediatric patients in non-outbreak settings. Furthermore, we evaluated the mortality associated with these infections.

## Methods

### Study selection

Three researchers (MF, MA, SK) searched the PubMed and EMBASE databases up to October 9^th^ 2015 for studies eligible for inclusion. The exact term used was “[ESBL OR (extended spectrum beta lactamase)] AND (pediatric OR paediatric OR neonat* OR infant* OR child*)”. The articles were searched rigorously by abstract and title and potential publications in English, French and Spanish were accessed in full text. Additionally, the references of the accessed studies were searched for eligible studies. The authors of the papers were contacted, when needed, for clarification. This systematic review and meta-analysis was designed according to the Preferred Reporting Items for Systematic Reviews and Meta-Analyses (PRISMA) guidelines [11] (S1 Checklist).

Studies were considered eligible when they reported the number of all laboratory-confirmed BSI cases (discussed below under *Definitions*) and the number of cases attributed to ESBL-PE. The age limit of 19 was used to define pediatric cases and each child could have suffered from one or multiple bloodstream infections during the study period. We excluded the studies that did not differentiate between specimen contamination and laboratory-confirmed infections, as defined by the as defined by the Centers for Disease Control and Prevention (CDC) [12]. Case reports, case series, as well as conference abstracts were also excluded. Studies that reported outbreaks and studies that implemented stewardship intervention periods were excluded in effort to avoid possible over or underestimation of ESBL-PE prevalence.

### Definitions

Clinically suspected BSIs were defined as cases where a blood culture was ordered based on symptomatology and clinical markers. Laboratory-confirmed BSIs (LCBSIS) were defined as clinically suspected cases where the blood culture showed significant pathogen growth that justified the need for treatment. Infections occurring in non-hospitalized children or within 48 hours of hospitalization were characterized as community-acquired, while those occurring after that time window were classified as nosocomial [13]. Healthcare-associated infections were defined as infections related to previous inpatient stay, indwelling medical devices, previous invasive procedures and surgeries and chemotherapy-induced neutropenia.[14]

### Data extraction and quality assessment

The primary outcome was the prevalence of ESBL-PE in pediatric LCBSIs and it was calculated by dividing the number of ESBL-PE BSI cases by the total number of LCBSI cases. As a secondary outcome, we calculated the prevalence among clinically suspected BSIs by dividing the number of ESBL-PE BSI cases by the total number of clinically suspected BSI cases, when this data was available. We also estimated the impact of ESBL-PE in all-cause BSI mortality.

As noted above, data extraction was performed by three individual researchers (MF, MA, SK) and discrepancies between them were resolved by consensus. We developed a spreadsheet that included the following data: study midyear and study duration, whether the study was prospective or retrospective, country and continent, age group of patients, hospital setting of the study, characteristics of patients in studies that selected a subgroup of hospitalized children, or in studies that had separate subpopulations, number of LCBSI cases, number of ESBL-PE LCBSI cases, ESBL- producing isolated species, ESBL microbiological method of detection. For studies that did not report their time frame, we assumed that the study period was 2 years prior to publication.

We evaluated the methodological quality of all included studies with the Newcastle–Ottawa Quality Assessment Scale (NOS),[15] a “star-based” rating system that consists of three parts (selection, comparability, and outcome). Studies received “stars” based on the representativeness of the exposed cohort, ascertainment of exposure, assessment of outcome, adequacy of follow-up time or outcomes to occur, and adequacy of follow-up of cohorts. Included studies could obtain a maximum of 5 stars because the parameters “selection of the non-exposed cohort”, “demonstrated that the outcome of interest was not present at the start of the study”, and “comparability between cohorts”, were not pertinent to our analysis. Studies that received at least 3 stars were considered adequate in quality (S1 Table).

### Data analysis

Since heterogeneity was expected because of the variable rates of ESBL-PE in different countries reported in the literature, the meta-analysis was performed using a random-effects model to estimate the pooled prevalence and the 95% confidence intervals (CI) [16]. To ensure proportionate weight distribution to studies presenting extreme prevalence (near 0 or 1), we applied the Freeman-Tukey arcsine methodology.[17] The heterogeneity of the studies was estimated by the tau-squared[18] and possible sources of heterogeneity were investigated by subgroup/sensitivity analysis following Knapp and Hartung approach.[19] We stratified the studies per region; due to aggregation of studies from India, these were separately analyzed from the rest of the Asian continent. The effect of small studies in publication bias was calculated by the Egger’s test.[20] The effect of ESBL-PE on mortality compared to non- ESBL-PE BSI cases was estimated using random effects meta-analysis and reported as unadjusted Risk Difference (RD) estimates and confidence intervals (95%CI). The time trend model for ESBL-PE infection was created by transforming the model coefficients to rates and plotting them against the midyear along with the observed prevalence rates [21]. Statistical analysis was performed using STATA v13 software package (STATA Corporation, College Station, TX). Statistical significance was set at 0.05 (two-tailed).

## Results

**Fig 1:**
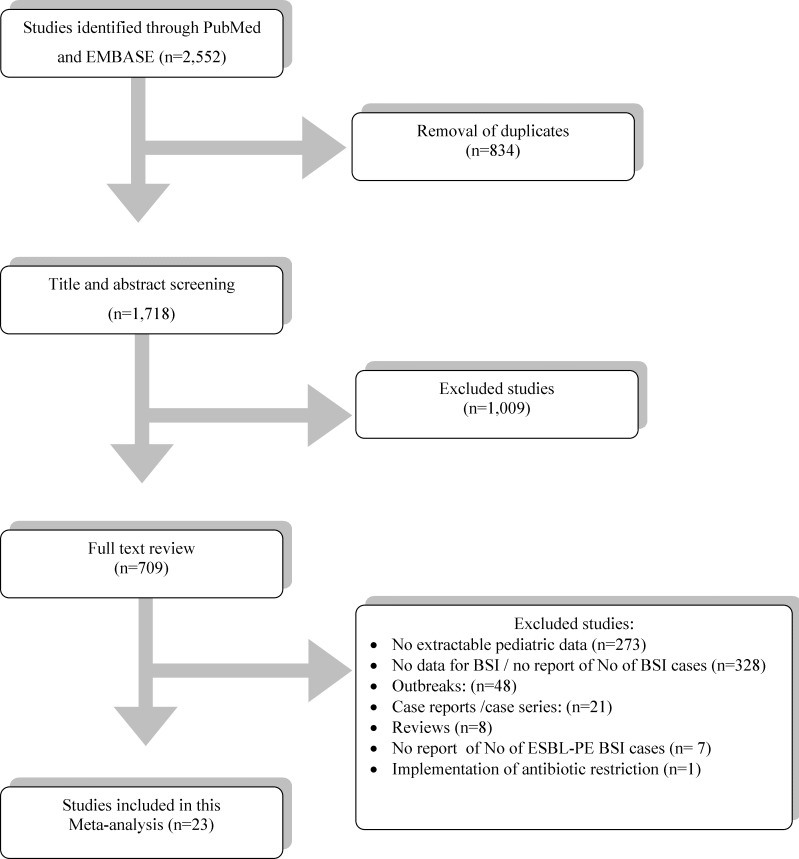
PRISMA flow diagram.

The database search yielded 1,718 non-duplicate abstracts. Among them, 1,009 were removed by title and abstract screening and 709 were accessed in full text. Among the 686 excluded studies, 273 did not report extractable pediatric data, 328 did not report data for BSI or did not report the exact number of BSI cases, 48 described outbreaks, 21 were case reports or case series, 8 were reviews, 7 did not specifically mention the number of ESBL-PE cases and 1 included data after implementation of antibiotic restriction. Twenty three studies met the inclusion criteria and were included in this meta-analysis. Screening of the reference lists did not yield additional papers. All studies were deemed of high quality (≥3 stars). The review process is depicted in Fig 1.

**Fig 2:**
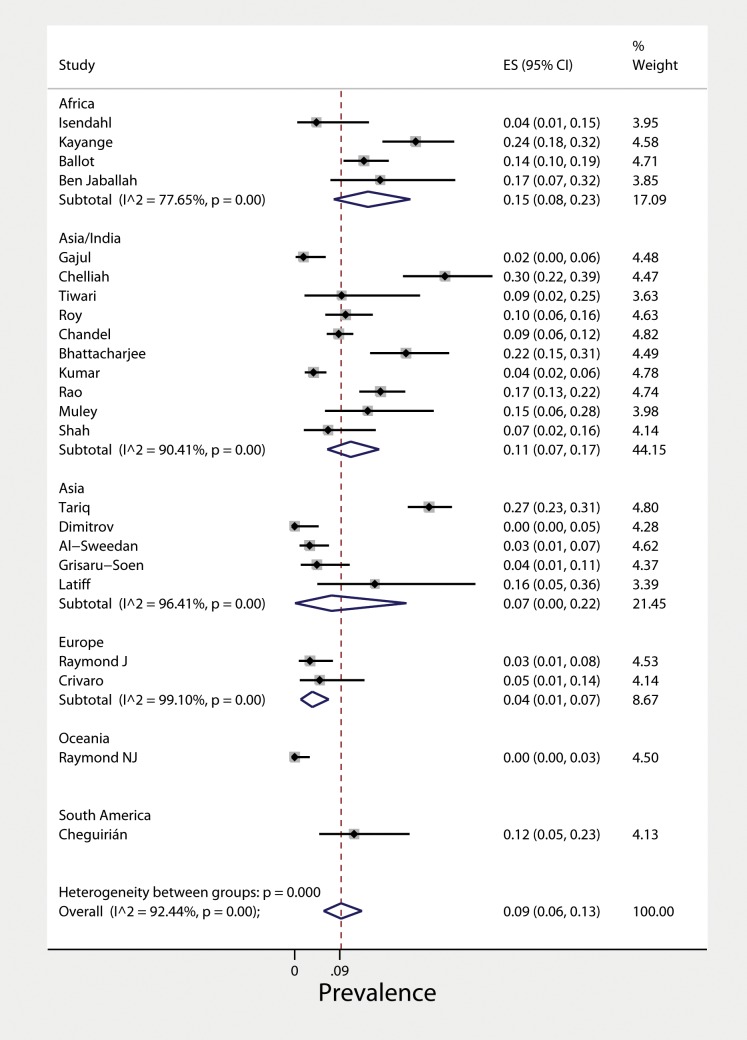
**Prevalence of ESBL-PE among laboratory-confirmed bloodstream infections in pediatric patients:** forest plot of included studies and geographical distribution.

**Fig 3:**
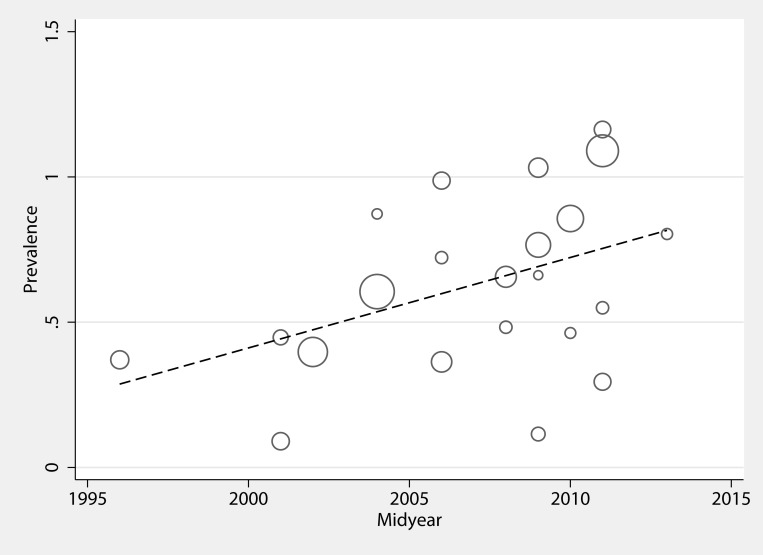
Time trend of ESBL-PE laboratory-confirmed bloodstream infections (1996–2013) depicting annual increase of 3.2%. Circles illustrate the estimates from each study, sized proportionately to the precision of each estimate. The fitted regression line is represented by study midyear.

The 23 included studies [14,22–43] reported data for a total 3,381 LCBSI cases, ranging from 1996 to 2013 (Table 1). Fourteen studies were prospective [14,23,25,26,28–30,32,33,35,37,40–42], whereas 9 were retrospective [22,24,27,31,34,36,38,39,43]. The pooled prevalence of ESBL-PE infection was 9% [95%CI (6, 13), τ^2^ = 0.09] with no evidence of small study effect across studies (Egger’s bias = -0.491, *P* = 0.440). Among them, 12 studies [22–25,29,32,34–36,40–42] provided data for 8,568 clinically suspected infections. Their pooled prevalence was 5% [95%CI (3, 7), τ^2^ = 0.03, (Egger’s bias = 0.859, *P* = 0.133)]. Regarding continent distribution, the pooled prevalence of LCBSIs was 15% in Africa (4 studies) [95%CI (8, 23)], 12% in South America (1 study) [95%CI (5, 23)], 11% in India (10 studies) [95%CI (7, 17)], 7% in the rest of Asia (5 studies) [95%CI (0, 22)], 4% in Europe (2 studies) [95%CI (1, 7)] and 0% in Oceania (1 study) [95%CI (0, 3)] (Fig 2). Interestingly, no studies from North America were identified. Comparing the rates from different regions did not yield statistically significant results (*P* = 0.081). The time trend plot we produced using the mid-years showed a statistically significant 3.2% annual increase in ESBL-PE LCBSIs (*P* = 0.04) (Fig 3). We have performed a sensitivity analysis, excluding a single study [29]that did not provide the timeframes of study conduction and found no significant time trend (*P =* 0.077)

**Table 1:** Summary of the 23 included studies.

Author	Country	Midyear^1^	Duration months^*2*^	Study type	Age^*2*^	Hospital setting^*3*^	Patients^*2*^^,^^*4*^	ESBL-PE	LCBSI Cases^*5*^	Prevalence %	Isolates^*2*^ ^*6*^	ESBL detection^*2*^^,^ ^*7*^
Africa												
Isendahl [32]	Guinea Bisau	2010	34	Prospective	0–5 years	Emergency department, tertiary hospital	N/A	2	46	4.35	K	VITEK 2, E test, Disk diffusion test (EUCAST) PCR. Identification: VITEK 2
Kayange [42]	Tanzania	2009	9	Prospective	0–28 days	Neonatal units, tertiary hospital	N/A	36	149	24.16	K, E and others	Screen: MacConkey agar CTX, Confirm: Double disk synergy test
Ballot [27]	South Africa	2009	13	Retrospective	0–28 days	Neonatal unit, tertiary hospital	N/A	34	246	13.82	K	N/A
Ben Jaballah [28]	Tunisia	2004	24	Prospective	0–15 years	PICU, tertiary hospital	Admitted >48 hours	7	41	17.07	K	N/A
Asia												
Tariq [34]	Afghanistan	2011	30	Retrospective	1 day-18 years	ICU and wards, Pediatric hospital	N/A	110	410	26.83	K, E, EB, SR and others	Screen: CTX, CAZ Confirm: Disk diffusion test
Dimitrov [43]	Kuwait	2009	96	Retrospective	N/A	Infectious diseases hospital	N/A	0	75	0	N/A	Screen: Disk diffusion test Confirm: E test
Latiff [39]	Malaysia	1999	12	Retrospective	9 months-17 years	Pediatric haematology oncology unit	Febrile neutropenic	4	25	16	K	N/A
Bhattacharjee [29]	India	2006	14	Prospective	0–28 days	NICU university hospital	N/A	26	117	22.22	K, E, P, A	Screen: Mueller Hinton Agar CTX, CAZ, Confirm: Disk diffusion test (CLSI), MIC reduction method
Chandel [41]	India	2004	36	Prospective	0–60 days	Multicentre: town and tertiary hospitals	N/A	42	478	8.79	K, E	Screen: CTX, CAZ, CFP Confirm: double disk synergy test CAZ, CTX±AMX/CLA
Chelliah [40]	India	2011	18	Prospective	0–28 days	NICU, Tertiary hospital	N/A	33	110	30	K, E and others	Screen: CTX, CAZ. Confirm: Disk diffusion test CAZ, CTX±CLA, MIC reduction. PCR
Gajul [22]	India	2011	25	Retrospective	0–28 days	NICU, Tertiary hospital	N/A	2	114	1.75	K	Screen: CPD, CFP, CTX CRO, ATM. Confirm: Disk diffusion test CAZ±CLA
Kumar [24]	India	2002	N/A	Retrospective	0–28 days	Multicenter: Neonatal Units	N/A	13	346	3.76	K	Double disk synergy test
Muley [36]	India	2013	N/A	Retrospective	0–28 days	NICU, Tertiary Hospital	N/A	7	48	14.58	K, E	Per CLSI criteria
Rao [23]	India	2010	N/A	Prospective	0–28 days	NICU, university hospital	N/A	48	280	17.14	K, E, EB, C, A	Disk diffusion test: CAZ±CLA
Roy [35]	India	2008	5	Prospective	0–28 days	Neonatal nurseries, Tertiary hospital	N/A	18	177	10.17	K,E	Screen Etest: CRO, CAZ, FEP Confirm Disk diffusion test CAZ, CTX±CLA
Tiwari [25]	India	2009	12	Prospective	1 day-10 years	Pediatric wards, University hospital	N/A	3	32	9.38	K, E	Double disk synergy test
Shah [37]	India	2011	2	Prospective	0–28 days	NICU, tertiary hospital	N/A	4	60	6.67	N/A	Per CLSI criteria
Grisaru-Soen [38]	Israel	2001	24	Retrospective	N/A	PICU, Tertiary Children’s Hospital	Admitted >48 h	4	90	4.44	K	N/A
Al-Sweedan [31]	Jordan	2006	60	Retrospective	0–17 years	University hospital	Admitted with febrile neutropenia	5	167	2.99	N/A	Disk diffusion test: CPD, CAZ, CTX±CLA
Europe												
Crivaro [30]	Italy	2008	60	Prospective	0–28 days	NICU, tertiary hospital	Admitted >48 hours, catheter associated	3	60	5	K	N/A
Raymond J [26]	Multinational	1996	6	Prospective	N/A	Multicenter: Pediatric and General hospitals	Admitted >48 hours	4	131	3.05	K	Double disk synergy test: CAZ, CRO, CTX, ATM ± AMX/CLA
Oceania												
Raymond NJ [14]	New Zealand	2001	12	Prospective	0–28 days	Multicenter: secondary and tertiary care hospitals	Healthcare -associated	0	50	0	N/A	Screen: VITEK Confirm: Double disk synergy test (Jarlier)
					1 month -19 years		Healthcare- associated	0	30	0	N/A	
							Community- acquired	0	40	0	N/A	
South America												
Cheguirián [33]	Argentina	2006	16	Prospective	1 month -15 years	Tertiary Children’s hospital	Admitted oncology patients Primary and secondary BSI	5	37	13.51	K, E, EB	VITEK Identification: VITEK, API
							Admitted oncology patients, CVC associated	2	22	9.09		

Country, midyear, study duration, study type, age group, hospital setting, patient characteristics, ESBL-PE cases, BSI cases, prevalence of ESBL-PE, ESBL-producing species isolated and methods of microbiological detection.

^1^ For studies that did not report their time frame, we assumed that the study period was 2 years prior to publication.

^2^ N/A: non- applicable.

^3^ NICU: Neonatal Intensive Care Unit, PICU: Pediatric Intensive Care Unit.

^4^ CVC: central venous catheter.

^5^LCBSIs: Laboratory-confirmed Bloodstream Infections.

^6^ A: *Acinetobacte*r spp, C: *Citrobacter* spp, E: *Escherichia*. *coli*, EB: *Enterobac*ter spp, K: *Klebsiella s*pp, SR: *Serratia s*pp.

^7^AMX: amoxicillin, ATM: aztreonam, CLA: clavulanic acid, CAZ: ceftazidime, FEP: cefepime, CRO: ceftriaxone, CFP: cefoperazone, CPD: cefpodoxime, CTX: cefotaxime.

Among the 23 studies included in our analysis, 16 studies provided data stratified by age: 12 [14,22–24,27,29,30,35–37,40,42] reported 1,757 neonatal LCBSIs with a pooled prevalence of 11% [95% CI (6, 17)], 4 studies [14,31,33,39] reported 321 infections in children older than 28 days with a pooled prevalence of 5% [95%CI (0, 14)]. The difference was not statistically significant (*P* = 0.499). The prevalence of LCBSIs among patients hospitalized in Neonatal Intensive Care Unit (NICU) was 11% (12 studies)[95%CI (4, 20)],[14,22–24,27,29,30,35–37,40,42] in the Pediatric Intensive Care Unit (PICU) 7% (2 studies) [95%CI (3, 13)][28,38] and in the Emergency Department 4% (1 study) [(95%CI (1, 15)] [32].

Five studies [14,26,28,30,38] provided data on 372 nosocomial LCBSIs with a pooled prevalence of 4% [95%CI (1, 9)], while 2 studies [14,32] provided data for 86 community-acquired BSIs with a pooled prevalence of 2% [95%CI (0, 6)], with no statistically significant difference (*P* = 0.71). Moreover, 4 studies [14,31,33,39] reported 244 healthcare-associated LCBSIs with a pooled prevalence of 5% [95%CI (0, 12)]. Of note is that there was no statistically significant difference in the rates between studies that reported the microbiologic method of detection (either phenotypic ESBL confirmatory tests [14,22–26,29,31,32,34–37,40–43] (τ^2^ = 0.10), or automatic methods [33],) and the studies that did not [27,28,30,38,39] (τ^2^ = 0.02), (*P* = 0.574). Similarly, there was no statistically significant difference in rates between studies that specified that a single LCBSI case per patient was recorded [13%, 95%CI (8, 19)] [22,23,29,31,32,34–37,40–42] and those that did not address this point [(6%, 95%CI (2, 10)] [14,24–28,30,33,38,39,43].

**Fig 4:**
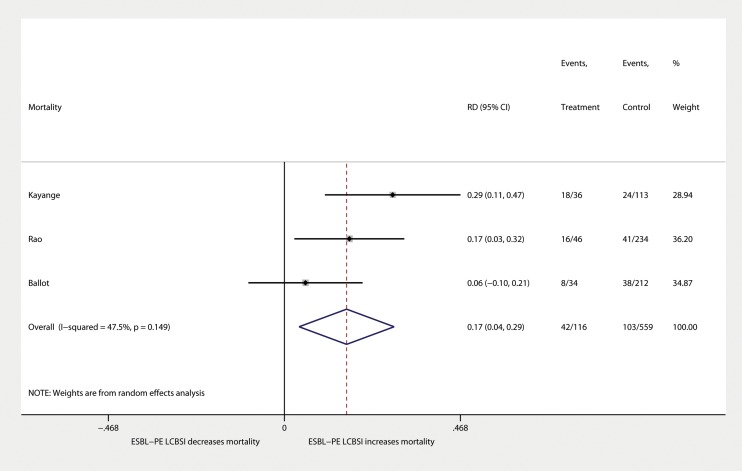
Mortality among neonates with ESBL-PE and non-ESBL-PE LCBSI. Forest plot of risk difference (RD).

Based on 3 studies [23,27,42] that recorded mortality data for 675 neonatal LCBSI cases, the pooled all-cause mortality rate among patients infected with ESBL-PE was 36% [95%CI (22, 51), τ^2^ = 0.04, (Egger’s bias = 0.46, *P* = 0.688)], compared to 18% among BSIs from all other pathogens [95%CI (15, 22), τ^2^ = 0, (Egger’s bias = 0.14, *P* = 0.003)], presenting a statistically significant difference in mortality risk (*P* = 0.01) [pooled RD = 16.5%, 95%CI = (3.9, 29.1), (Egger’s bias = 9.16, *P* = 0.491)] (Fig 4).

## Discussion

We highlight the emergence of ESBL-PE in the pediatric patients with BSI. We found a considerable rate of 9% among all LCBSI cases; the incidence is even higher in Africa, South America and India and is increasing over time. Importantly, these infections appear to be more common among neonates while the mortality rate among neonates with BSI due to ESBL-PE was 36% and was significantly higher compared to those infected with other pathogens.

The reported prevalence of ESBL-PE in pediatric BSIs shows the global emergence of these resistant infections and our results are comparable to the rates of ESBL-PE among pediatric bloodstream Enterobacteriaceae, observed in different regions that range from 25% to 70% in endemic areas [6,8,44–48]. Moreover, we estimated that the annual increase is 3.2%. Interestingly, a study documenting 368,398 pediatric Enterobacteriaceae isolates revealed that the prevalence of ESBL-PE has tripled during the period between 1990 and 2011 [3]. Another study calculated the incidence of pediatric ESBL-PE among 2,697 *Escherichia coli*, and *Klebsiella* spp. to be 2.2 times greater in the last 2.5 years, compared to the first 30 months of the study period (2003–2007) [49]. Further studies are needed to evaluate the current burden as well as why current preventive measures are failing to control the dissemination of these important resistant pathogens. For example, their rise is associated with selective pressure exerted by antimicrobial agents [50], thus antimicrobial stewardship protocols might help control their spread and should be specifically evaluated in this patient population.

Regarding age distribution, ESBL-PE infections were frequently encountered in neonatal units. A recent case-control study involving 110 pediatric patients with BSIs from ESBL-PE found that newborns are more likely to suffer from ESBL-PE BSIs compared to older children, irrespectively of concomitant co-morbidities [7]. The neonatal immune system requires 5–7 days to develop and during this time, newborns depend on the innate immune response and passive immunization by the mother [51]. Moreover, ESBL-PE infections have been associated with prematurity [52], further underlining the role of immaturity of the immune system in these infections.

Importantly, our analysis showed a higher mortality among neonates infected with ESBL-PE. High fatality rates and have been observed in neonatal infections with ESBL-PE [47,53–55] and our analysis demonstrates this difference with statistically significant results. In a study performed at the NICU of a tertiary hospital higher mortality was documented among BSI cases with ESBL–PE (23.6 vs. 4.0%), though the difference was not statistically significant. [54] Also, in a prospective study that included 400 bloodstream pathogens, more than 60% of neonates infected with ESBL- PE died in contrast with 35.7% of those infected with other isolates [47].

Regarding strengths, our analysis included studies in 3 major languages and highlights the emergence of ESBL-PE in the understudied pediatric population. Regarding limitations of our meta-analysis, data on consumption of antimicrobial agents prior to blood culture were not provided in most studies and the use of antimicrobial agents has been associated with higher ESBL-PE rates [45]. Also, we found no significant difference between different hospital settings and this may be affected by the small sample of studies reporting stratified data on this parameter. Regarding mortality rates, data were available only for neonatal infections and further studies are needed to estimate rates in the other age groups. Further stratification to account for patient comorbidities and gestational age was not possible based on the available data. Finally, five studies did not report the method used for ESBL isolation while ten studies did not report the exclusion of recurrent infections, but, as shown above, this did not significantly affect our results.

In conclusion, in the pediatric population, BSIs caused by ESBL-PE are increasing in frequency and, at least among neonates are associated with significantly higher mortality rates. Preventive and screening protocols, development of rapid diagnostics and the appropriate treatment of children at high risk should be evaluated. For this, studies are warranted to identify potential risk factors and establish a well-demarcated high-risk group and address the lack of data from North America.

## Supporting Information

S1 Checklist PRISMA checklist. (DOCX)

S1 Table Quality assessment of eligible studies. (DOCX)
