# Supplementary material for: Prevalence of ESBL-Producing Enterobacteriaceae in Pediatric Bloodstream Infections: A Systematic Review and Meta-Analysis
Source: PLoS One. 2017 Jan 31;12(1):e0171216. doi: 10.1371/journal.pone.0171216 (PMC5283749; doi:10.1371/journal.pone.0171216)
Supplement: S1 Table — (DOCX) [file pone.0171216.s002.docx]

| Study | Selection | | | | Comparability | Outcome | | | score |
| --- | --- | --- | --- | --- | --- | --- | --- | --- | --- |
|  | Representativeness of the exposed cohort | Selection of the non-exposed cohort | Ascertainment of exposure | Demonstration that outcome of interest was not present at start of study | Comparability of cohorts on the basis of the design or analysis | Assessment of outcome | Follow-up long enough for outcomes to occur | Adequacy of follow-up of cohorts |  |
| Al-Sweedan | - | N/A | ★ | N/A | N/A | ★ | ★ | ★ | 4 |
| Ballot | ★ | N/A | ★ | N/A | N/A | - | ★ | ★ | 4 |
| Ben Jaballah | ★ | N/A | ★ | N/A | N/A | - | ★ | ★ | 5 |
| Bhattacharjee | ★ | N/A | ★ | N/A | N/A | ★ | ★ | ★ | 5 |
| Chandel | ★ | N/A | ★ | N/A | N/A | ★ | ★ | ★ | 5 |
| Cheguirián | - | N/A | ★ | N/A | N/A | ★ | ★ | ★ | 4 |
| Chelliah | ★ | N/A | ★ | N/A | N/A | ★ | ★ | ★ | 5 |
| Crivaro | ★ | N/A | ★ | N/A | N/A | - | ★ | ★ | 4 |
| Dimitrov | ★ | N/A | ★ | N/A | N/A | ★ | ★ | ★ | 5 |
| Gajul | ★ | N/A | ★ | N/A | N/A | ★ | ★ | ★ | 5 |
| Grisaru-Soen | ★ | N/A | ★ | N/A | N/A | - | ★ | ★ | 4 |
| Isendahl | ★ | N/A | ★ | N/A | N/A | ★ | ★ | ★ | 5 |
| Kayange | ★ | N/A | ★ | N/A | N/A | ★ | ★ | ★ | 5 |
| Kumar | ★ | N/A | ★ | N/A | N/A | ★ | ★ | ★ | 5 |
| Latiff | - | N/A | ★ | N/A | N/A | - | ★ | ★ | 3 |
| Muley | ★ | N/A | ★ | N/A | N/A | ★ | ★ | ★ | 5 |
| Rao | ★ | N/A | ★ | N/A | N/A | ★ | ★ | ★ | 5 |
| Raymond J | ★ | N/A | ★ | N/A | N/A | ★ | ★ | ★ | 5 |
| Raymond NJ | ★ | N/A | ★ | N/A | N/A | ★ | ★ | ★ | 5 |
| Roy | ★ | N/A | ★ | N/A | N/A | ★ | ★ | ★ | 5 |
| Shah | ★ | N/A | ★ | N/A | N/A | ★ | ★ | ★ | 5 |
| Tariq | ★ | N/A | ★ | N/A | N/A | ★ | ★ | ★ | 5 |
| Tiwari | ★ | N/A | ★ | N/A | N/A | ★ | ★ | ★ | 5 |

**S1 Table:** Quality assessment of eligible studies
